# Unipolar atrial electrogram morphology is affected by age: evidence from high-resolution epicardial mapping

**DOI:** 10.1080/07853890.2023.2193426

**Published:** 2023-05-17

**Authors:** Ziliang Ye, Mathijs S. van Schie, Annejet Heida, Lianne N. van Staveren, Frank R. N. van Schaagen, Yannick J. H. J. Taverne, Natasja M. S. de Groot

**Affiliations:** a Department of Cardiology, Erasmus Medical Center, Rotterdam, The Netherlands; b Translational Cardiothoracic Surgery Research Lab, Department of Cardiothoracic Surgery, Erasmus Medical Center, Rotterdam, The Netherlands

**Keywords:** Unipolar atrial electrogram, ageing, coronary artery bypass grafting, sinus rhythm, epicardial mapping

## Abstract

**Background:**

It is unknown which features of unipolar atrial electrogram (U-AEGM) morphology are affected by ageing and whether age-related changes in U-AEGM morphology are equally distributed throughout the right and left atria.

**Patients and methods:**

Epicardial high-resolution mapping was performed in patients undergoing coronary artery bypass grafting surgery during sinus rhythm (SR). Mapping areas include the right atrium (RA), left atrium (LA), pulmonary vein area (PVA) and Bachmann’s bundle (BB). Patients were categorized into a young (age < 60) and aged (age ≥ 60) group. U-AEGM were classified as single potentials (SPs, one deflection), short double potentials (SDPs, deflection interval ≤ 15ms), long double potentials (LDPs, deflection interval > 15ms) and fractionated potentials (FPs, ≥3 deflections).

**Results:**

A total of 213 patients (age: 67 (59–73) years; young group *N* = 58, aged group *N* = 155) were included. Only at BB, the proportion of SPs (*p* = 0.007) was significantly higher in the young group, while the proportion of SDPs (*p* = 0.051), LDPs (*p* = 0.004) and FPs (*p* = 0.006) was higher in the aged group. After adjusting for potential confounders, older age was associated with a reduction in SPs [regression coefficient (β): −6.33, 95% confident interval (CI): −10.37 to −2.30] at the expense of an increased proportion of SDPs (β: 2.49, 95% CI: 0.09 to 4.89), LDPs (β: 1.94, 95% CI: 0.21 to 3.68) and FPs (β: 1.90, 95% CI: 0.62 to 3.18).

**Conclusions:**

Age-related remodeling particularly affects BB as indicated by the decreased amount of non-SP at this location in the elderly.

**Key Messages**
- Ageing preferentially affects the morphology of unipolar atrial electrograms recorded at Bachmann’s bundle.
- At Bachmann’s bundle, the proportion of short double-, long double- and fractionated potentials increase during ageing at the expense of a decrease in the proportion of single potentials, reflecting aggravation of abnormalities in conduction.
- The increase in abnormal unipolar atrial electrograms at Bachmann’s bundle during ageing supports the concept that Bachmann’s bundle may play an important role in development of age-related arrhythmias such as atrial fibrillation.

## Introduction

Ageing is one of the principal risk factors for development of atrial fibrillation (AF) [1–3]. The reported prevalence of AF ranges between 0.12% and 0.16% in subjects younger than 49 years and increases up to 17% above 80 years or older [4]. Although ageing is currently one of the most important factors resulting in worldwide health issues such as AF [5], data on age-related changes in atrial electrophysiology is scarce.

In canine models, Anyukhovsky et al. [1] demonstrated that the average action potential duration was significantly longer in the right and left atria of elderly dogs compared to adult dogs. Also, action potential duration heterogeneity was more pronounced in atrial tissues of old dogs. In humans, endocardial mapping studies revealed that ageing is associated with slowing of conduction at the right atrium (RA) and the presence of diffusely spread low-voltage areas [6]. Recently, Van der Does et al. [7] revealed that ageing was associated with more conduction disorders, especially at Bachmann’s bundle (BB) and the RA.

However, current data on age-related changes in electrogram (EGM) morphology is limited to the RA. Kistler et al. [6] demonstrated in 41 patients that ageing was related to a larger number of double and fractionated potentials along the crista terminalis. However, it is unknown which features of EGM morphology are affected by ageing and whether the age-related changes in EGM morphology are equally distributed throughout the right and left atria (LA). The purpose of this study is therefore to investigate the influence of age on unipolar atrial EGM (U-AEGM) morphology recorded from the RA and LA including BB in a large cohort of patients undergoing cardiac surgery.

## Methods

### Study population

The study population included adult participants, who underwent coronary artery bypass grafting (CABG) surgery at Erasmus Medical Center in Rotterdam between March 2012 to August 2020 (8, 9). This project has been approved by the institutional medical ethics committee (MEC2010-054/MEC2014-393) [8,9], and written informed consent was obtained from all participants before enrolling. Baseline demographic and clinical profiles (e.g. age, gender, body mass index and underlying heart diseases) were attained from the hospital’s electronic medical system. None of the patients included in this study had a history of AF. All included participants were grouped into a young (age < 60) and aged (age ≥ 60) group, which was based on previous studies [10,11].

### Mapping procedure

**Figure 1.**
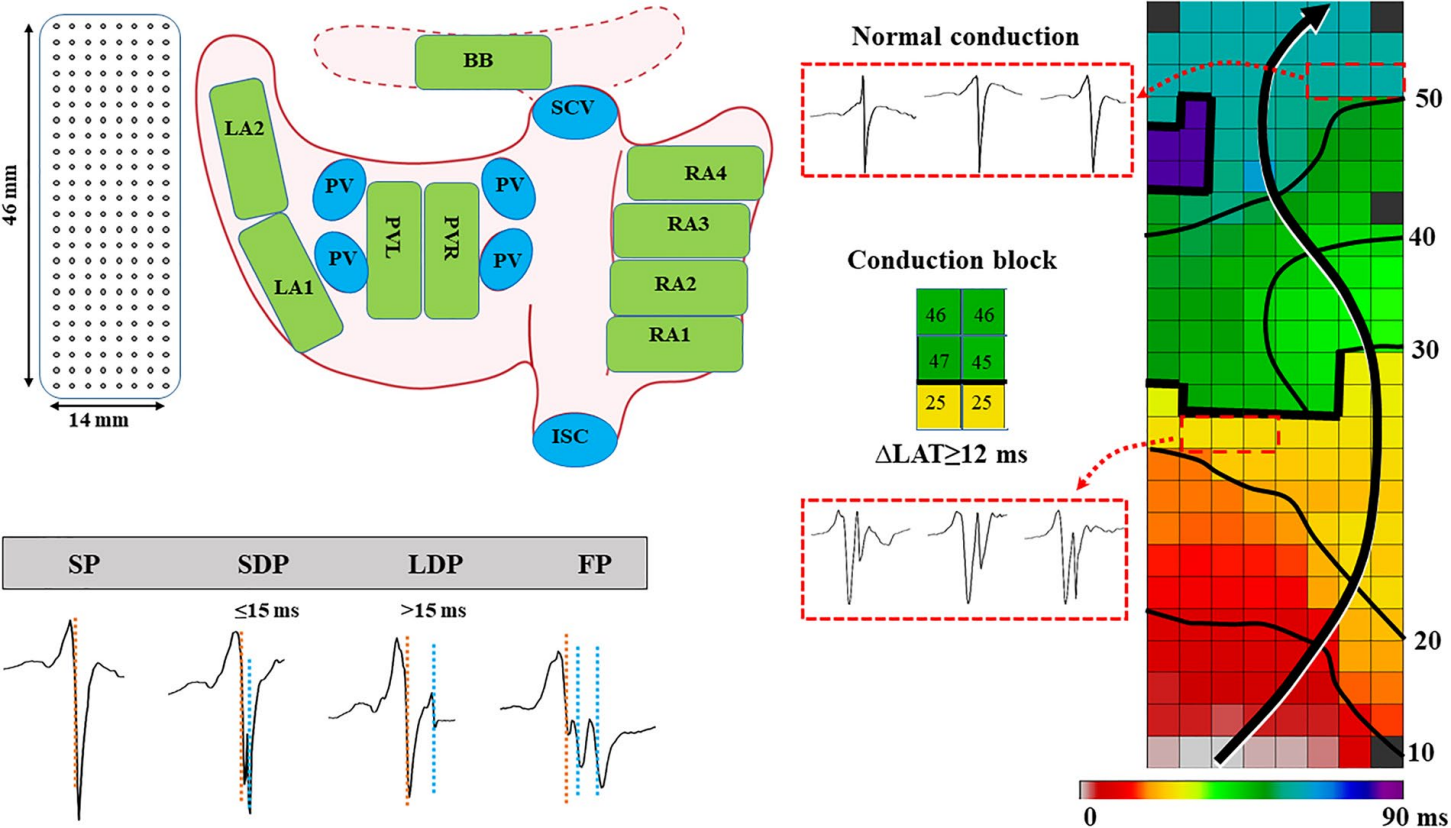
The upper left panel shows a schematic presentation of the 192-unipolar electrode array on the RA, LA and BB. The upper right panel demonstrates a typical color-coded activation map demonstrating lines of conduction block, defined as local activation time differences between adjacent electrodes of ≥12ms; corresponding long double potentials are shown outside the activation map. Isochronal lines (thin black lines) are drawn at 10-millisecond intervals, and the black arrow indicates the propagation direction of the SR wavefront. IVC: inferior vena cava; SVC: superior vena cava; RA: right atrium; BB: Bachmann’s bundle; LA: left atrium; PV(A) : pulmonary vein (area); SP: single potential; SDP: short double potential; LDP: long double potential; FP: fractionated potential.

As described in our previous studies, epicardial high-resolution mapping was carried out before extracorporeal circulation [8,9]. In short, a temporary bipolar pacemaker lead was sutured on the free wall of the RA and served as a reference electrode, and a steel wire was fixed on subcutaneous tissue of the thoracic cavity as a neutral electrode. A 128- or 192- electrode array (electrode diameter: 0.65 or 0.45 mm, interelectrode distances: 2.0 mm) was used for epicardial mapping of the atria (upper left panel of Figure 1). According to a predefined mapping scheme (upper left panel of Figure 1), the electrode array was moved along an imaginary line with a fixed anatomical orientation for mapping, covering the entire epicardial atrial surface [including RA, BB, pulmonary vein area (PVA), and LA]. Mapping of the RA was performed from the inferior to superior caval vein perpendicular to the terminal crest. The PVA was accessed through the oblique sinus along the boundary of the left and right PV. Mapping of the LA was performed from the lower border of the left inferior PV towards the LA appendage. Additionally, BB was mapped from the tip of the LA appendage across the top of the LA and RA, behind the aorta towards the superior cavo-atrial junction. Five seconds of sinus rhythm (SR) was recorded at each mapping location, with a surface electrocardiogram (ECG) lead, a bipolar reference EGM, and all unipolar epicardial EGMs. Data were processed by amplification (gain 1,000), filtering (bandwidth 0.5–400 Hz), sampling (1 kHz) and analog-to-digital conversion (16 bits), and then saved on a hard disk.

### Data analysis

Customized software was used to semi-automatically analyze U-AEGM morphology. U-AEGMs with injury potentials or recording sites with ≥25% missing U-AEGMs were excluded. In addition, U-AEGMs recorded during premature atrial beats or aberrant beats were eliminated. Under the premise that the deflection amplitude was at least twice the signal-to-noise ratio, the steepest negative slope of U-AEGMs was marked as the local activation time (LAT). All U-AEGM annotations were manually inspected by two researchers with consensus, and color-coded activation maps were reconstructed using the LATs on each electrode (upper right panel of Figure 1).

Consistent with Konings et al. [12], U-AEGMs were categorized as single potentials (SPs), short double potentials (SDPs), long double potentials (LDPs) and fractionated potentials (FPs). In brief, SPs only consist of a single negative deflection; SDPs and LDPs contain two negative deflections with time interval between deflections of respectively ≤15ms and >15 ms; FPs were defined as ≥3 deflections. Examples of the different U-AEGM morphologies are shown in the lower left panel of Figure 1.

### Statistical analysis

A Shapiro–Wilk test was applied to inspect the distribution of continuous variables. Continuous variables conforming to a normal distribution were described as mean ± standard deviation (SD), and the differences were compared using an independent t-test. Skewed distributed variables were described as median and interquartile range (IQR), and a Mann–Whitney U test was performed to compare differences between groups. Categorical variables were described as the number and percentage, and the differences were assessed by χ^2^ test. A P-value (two-sides) less than 0.05 indicates significant difference.

Univariable linear regression analysis was performed to investigate which variables were associated with different U-AEGM morphologies (including SP, SDP, LDP, and FP). We further explored the independent relationship between age (independent variable) and different U-AEGMs morphologies (dependent variables) using a multivariable linear regression model. In this process of multivariable linear regression analysis, two models were performed: model I, covariates related to different U-AEGM morphologies in univariable linear regression analysis were adjusted; model II, all covariates, including body mass index, gender, hypertension, dyslipidemia, diabetes mellitus, myocardial infarction, left ventricular function, left atrial dilatation, and medication, were adjusted. Results were described as regression coefficient (β) and 95% confidence interval (CI).

Furthermore, a generalized linear model (GLM) was performed to visualize the independent relationship between age (considering as continuous variable) and different U-AEGM morphologies (considering as continuous variables), with the same adjustment for covariates in model I and model II. R software (version 4.1.3) and IBM SPSS Statistics (version 28) were used to analyze the data.

## Results

### Study population

A total of 213 patients with an age ranging from 37 to 84 years [median age: 67 (59–73), 85.45% male) were enrolled. This study population was categorized into a young (age < 60 years, *N* = 58, 82.76% male) and aged (age ≥ 60 years, *N* = 155, 86.45% male) group. Except for age [young: 54 (50–57) years versus aged: 70 (66–75) years, *p* < 0.001] and BMI [young: 29.5 kg/m^2^ (26.3–33.3) versus aged: 27.4 kg/m^2^ (25.2–30.3), *p* = 0.004], baseline characteristics of these 2 groups did not differ (Table 1).

**Table 1.** Baseline characteristics of the study population.

Variables	Overall	Young Group (age < 60 years)	Aged Group (age ≥ 60 years)	*p* Value
N	213	58	155	
Age, years, median (IQR)	67(59, 73)	54(50, 57)	70(66, 75)	<0.001
Male, *N* (%)	182(85.45)	48(82.76)	134(86.45)	0.644
BMI, median (IQR)	27.7(25.5, 31.2)	29.5(26.3, 33.3)	27.4(25.2, 30.3)	0.004
Hypertension, *N* (%)	132(61.97)	35(60.34)	97(62.58)	0.888
Dyslipidemia, *N* (%)	93(43.66)	26(44.83)	67(43.23)	0.956
Diabetes mellitus, *N* (%)	74(34.74)	17(29.31)	57(36.77)	0.392
Myocardial infarction, *N* (%)	105(49.30)	30(51.72)	75(48.39)	0.780
Left ventricular function, *N* (%)				0.914
Normal (EF >55%)	160(75.12)	44(75.86)	116(74.84)	
Mild impairment (EF 46%–55%)	46(21.60)	12(20.69)	34(21.94)	
Moderate impairment (EF 36%–45%)	6(2.82)	2(3.45)	4(2.58)	
Severe impairment (EF <35%)	1(0.47)	0(0.00)	1(0.65)	
Left atrial dilatation > 45 mm, *N* (%)	24(11.27)	5(8.62)	19(12.26)	0.673
ACEI/ARB/AT2 antagonist, *N* (%)	147(69.34)	40(68.97)	107(69.48)	1
Statin, *N* (%)	188(88.26)	49(84.48)	139(89.68)	0.418
Digoxin, *N* (%)	2(0.94)	0(0.00)	2(1.29)	0.943
Class I, *N* (%)	0(0.00)	0(0.00)	0(0.00)	NA
Class II, *N* (%)	170(79.81)	51(87.93)	119(76.77)	0.107
Class III, *N* (%)	3(1.41)	2(3.45)	1(0.65)	0.372
Class IV, *N* (%)	11(5.16)	2(3.45)	9(5.81)	0.730

IQR: interquartile range; *N*: number; BMI: body mass index; EF: ejection fraction; ACEI: angiotensin-converting enzyme inhibitors; ARB: angiotensin receptor blockers; AT2: angiotensin type 2 receptor; NA: not available.

### U-AEGM database

A total of 2,007,403 U-AEGMs (9,424 ± 2,818 per patient) with a mean SR cycle length of 886 ± 166 ms were recorded. The number of U-AEGMs obtained from the RA, BB, PVA and LA area was respectively 938,093 (46.73%), 227,465 (11.33%), 445,334 (22.19%) and 396,511 (19.75%). Characteristics of U-AEGMs recorded from all patients for every atrial region separately are shown in Supplemental Table S1. At each atrial region, the proportion of SPs was the largest (RA: 82.49%, BB: 79.76%, PVA: 83.58% and LA: 81.79%), whereas the proportion of FPs was the smallest (RA: 2.33%, BB: 2.60%, PVA: 1.52% and LA: 1.97%).

### Impact of ageing on U-AEGM morphology

**Figure 2.**
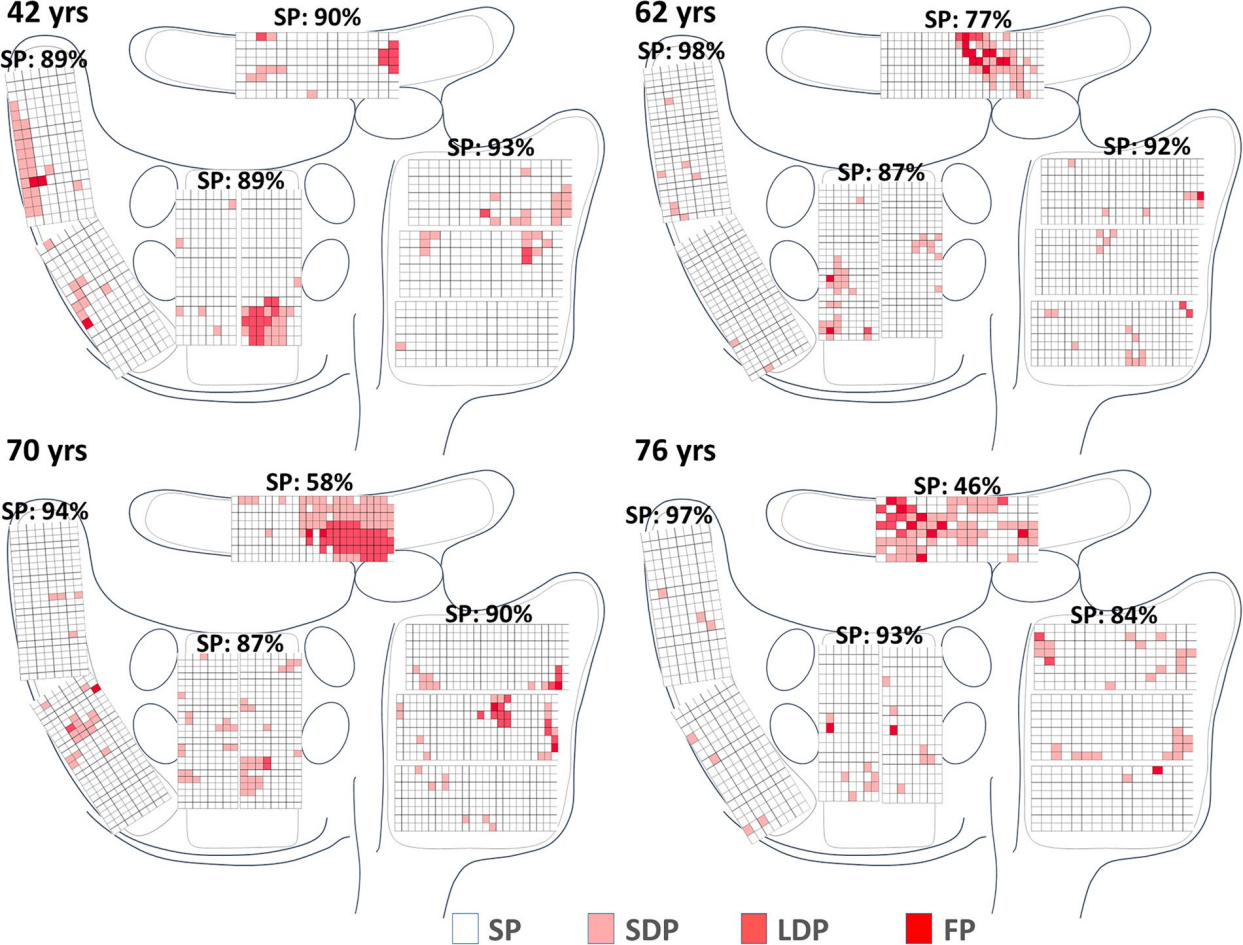
U-AEGM type maps demonstrating the proportions of the different U-AEGM morphologies, obtained from four representative patients of different ages ranging from 42 to 76 years. These maps clearly show that the proportion of SDPs, LDPs and FPs at BB is higher in the oldest patient. U-AEGM: unipolar, unipolar atrial electrogram; SP: single potential; SDP: short double potential; LDP: long double potential; FP: fractionated potential; BB: Bachmann’s bundle.

Figure 2 shows a color-coded distribution of SPs, SDPs, LDPs and FPs measured at all mapping areas in four typical patients of various ages (42-, 62-, 70- and 76-year-old). These maps show that at all areas, but particularly at BB, there are age-related changes in U-AEGM morphology consisting of an increase in non-SP.

The impact of ageing on U-AEGM morphology in the different areas in the entire study population is summarized in Table 2. In the aged group, only at BB the proportion of SPs was significantly lower compared to the young group (*p* = 0.007) whereas the proportions of SDPs (border significant, *p* = 0.051), LDPs (*p* = 0.004) and FPs (*p* = 0.006) were higher. However, an increased age did not have significant impact on U-AEGM morphology recorded from the RA, PVA and LA.

**Table 2.** Comparison of U-AEGMs characteristics between young and aged group.

	Young group (age < 60 years)	Aged group (age ≥ 60 years)	*p* Value
**BB**			
SPs, %, median (IQR)	84.1 (76.6, 91.3)	79.0 (71.2, 87.2)	0.007
SDPs, %, median (IQR)	9.9 (6.4, 15.8)	12.8 (8.5, 17.5)	0.051
LDPs, %, median (IQR)	1.6 (0.2, 5.3)	4.3 (1.3, 9.4)	0.004
FPs, %, median (IQR)	0.8 (0.3, 2.1)	1.6 (0.7, 3.9)	0.006
**LA**			
SPs, %, median (IQR)	82.1 (76.9, 89.4)	82.8 (75.9, 88.6)	0.617
SDPs, %, median (IQR)	12.0 (9.1, 17.3)	11.3 (8.3, 16.4)	0.512
LDPs, %, median (IQR)	0.8 (0.1, 3.6)	1.5 (0.6, 4.9)	0.150
FPs, %, median (IQR)	1.0 (0.3, 2.5)	1.2 (0.4, 2.5)	0.767
**PV**			
SPs, %, median (IQR)	84.4 (74.6, 89.0)	86.0 (77.9, 90.6)	0.180
SDPs, %, median (IQR)	11.3 (8.4, 17.3)	10.3 (6.8, 16.7)	0.231
LDPs, %, median (IQR)	1.3 (0.3, 5.2)	1.4 (0.3, 4.0)	0.978
FPs, %, median (IQR)	0.7 (0.2, 2.7)	0.8 (0.2, 1.9)	0.961
** RA**			
SPs, %, median (IQR)	83.3 (77.4, 88.4)	84.0 (77.9, 89.9)	0.511
SDPs, %, median (IQR)	10.2 (6.6, 12.1)	9.6 (5.8, 13.1)	0.379
LDPs, %, median (IQR)	4.1 (2.0, 6.6)	4.2 (1.7, 6.9)	0.727
FPs, %, median (IQR)	1.8 (0.6, 3.4)	1.4 (0.4, 2.9)	0.290

U-AEGMs: unipolar atrial electrograms; SPs: single potentials; SDPs: short double potentials; LDPs: long double potentials; FPs: fractionated potentials; IQR: interquartile range; BB: Bachmann’s bundle; LA: left atrium; PV: pulmonary vein; RA: right atrium.

### Age-related changes in BB U-AEGM morphology

Univariable predictors of age-related changes in U-AEGMs at BB with their respective β (95% CI) are summarized in Table 3. In univariable analyses, ageing was associated with a decrease in the proportion of SPs (β = −5.43, *p* = 0.006), and an increase in the proportions of LDPs (β = 1.90, *p* = 0.024) and FPs (β = 1.71, *p* = 0.005). Furthermore, the use of ACEI/ARB/AT2 antagonists was associated with an increase in the proportion of SPs (β = 4.14, *p* = 0.032) and a reduction in the proportion of LDPs (β = −1.88, *p* = 0.020); male (β = −3.46, *p* = 0.022) was associated with a decrease in the proportion of SDPs, while LA dilatation (β = 4.18, *p* = 0.024) was correlated with an increased SDP proportion. Additionally, we also found that digoxin usage was associated with a higher proportion of LDPs (β = 7.89, *p* = 0.040).

**Table 3.** Variables related to BB U-AEGM morphologies in univariable linear regression analysis.

SPs			
Variables	β	95% CI	p value
**Aged (age ≥60 years)**	**−5.43**	−**9.27 to −1.58**	**0.006**
Male	2.47	−2.42 to 7.36	0.321
BMI	−0.14	−0.57 to 0.30	0.542
Hypertension	−1.17	−4.77 to 2.43	0.522
Dyslipidemia	−0.42	−3.95 to 3.12	0.817
Diabetes mellitus	−2.25	−5.91 to 1.41	0.228
Myocardial infarction	0.12	−3.38 to 3.63	0.945
Left ventricular function			
Normal (EF >55%)	Ref	Ref	Ref
Mild impairment (EF 46%–55%)	0.07	−4.24 to 4.38	0.975
Moderate impairment (EF 36%–45%)	−4.10	−14.59 to 6.40	0.442
Severe impairment (EF <35%)	4.44	−20.87 to 29.74	0.730
Left atrial dilatation > 45 mm	−2.11	−8.15 to 3.93	0.492
**ACEI/ARB/AT2 antagonist**	**4.14**	**0.37 to 7.91**	**0.032**
Statin	2.22	−3.33 to 7.78	0.431
Digoxin	−13.25	−31.02 to 4.53	0.143
Class I	–	–	–
Class II	2.49	−1.97 to 6.95	0.273
Class III	−8.77	−23.35 to 5.80	0.237
Class IV	−4.90	−13.02 to 3.22	0.236
**SDPs**			
Variables	β	95% CI	P value
Aged (age ≥60 years)	1.82	−0.55 to 4.19	0.132
**Male**	−**3.46**	−**6.40 to −0.51**	**0.022**
BMI	0.14	−0.12 to 0.41	0.284
Hypertension	1.62	−0.56 to 3.81	0.144
Dyslipidemia	−0.08	−2.23 to 2.07	0.944
Diabetes mellitus	2.19	−0.02 to 4.41	0.053
Myocardial infarction	−0.24	−2.38 to 1.89	0.822
Left ventricular function			
Normal (EF >55%)	Ref	Ref	Ref
Mild impairment (EF 46%–55%)	−0.21	−2.82 to 2.41	0.875
Moderate impairment (EF 36%–45%)	4.26	−2.11 to 10.64	0.189
Severe impairment (EF <35%)	2.90	−12.47 to 18.28	0.710
**Left atrial dilatation > 45 mm**	**4.18**	**0.56 to 7.80**	**0.024**
ACEI/ARB/AT2 antagonist	−1.81	−4.11 to 0.49	0.123
Statin	−1.04	−4.43 to 2.35	0.546
Digoxin	3.33	−7.55 to 14.21	0.547
Class I	–	–	–
Class II	0.71	−3.44 to 2.01	0.605
Class III	8.36	−0.47 to 17.20	0.063
Class IV	4.09	−0.84 to 9.03	0.104
**LDPs**			
Variables	β	95% CI	P value
**Aged (age ≥60 years)**	**1.90**	**0.25 to 3.54**	**0.024**
Male	0.74	−1.34 to 2.82	0.486
BMI	−0.04	−0.23 to 0.14	0.660
Hypertension	−0.36	−1.89 to 1.17	0.640
Dyslipidemia	0.44	−1.06 to 1.94	0.565
Diabetes mellitus	−0.05	−1.61 to 1.51	0.951
Myocardial infarction	−0.23	−1.72 to 1.26	0.762
Left ventricular function			
Normal (EF >55%)	Ref	Ref	Ref
Mild impairment (EF 46%–55%)	−0.11	−1.94 to 1.72	0.907
Moderate impairment (EF 36%–45%)	−0.08	−4.54 to 4.38	0.973
Severe impairment (EF <35%)	−4.94	−15.69 to 5.81	0.366
Left atrial dilatation > 45 mm	−2.17	−4.73 to 0.39	0.096
**ACEI/ARB/AT2 antagonist**	**−1.88**	**−3.47 to −0.29**	**0.020**
Statin	−0.53	−2.89 to 1.83	0.659
**Digoxin**	**7.89**	**0.38 to 15.41**	**0.040**
Class I	–	–	–
Class II	−1.47	−3.36 to 0.42	0.127
Class III	0.57	−5.65 to 6.78	0.857
Class IV	0.57	−2.90 to 4.03	0.748
**FPs**			
Variables	β	95% CI	P value
**Aged (age ≥60 years)**	**1.71**	**0.52 to 2.89**	**0.005**
Male	0.25	−1.26 to 1.76	0.742
BMI	0.03	−0.10 to 0.17	0.639
Hypertension	−0.09	−1.20 to 1.02	0.874
Dyslipidemia	0.05	−1.03 to 1.14	0.922
Diabetes mellitus	0.10	−1.03 to 1.23	0.861
Myocardial infarction	0.35	−0.73 to 1.43	0.524
Left ventricular function			
Normal (EF >55%)	Ref	Ref	Ref
Mild impairment (EF 46%–55%)	0.25	−1.08 to 1.58	0.711
Moderate impairment (EF 36%–45%)	−0.08	−3.32 to 3.15	0.959
Severe impairment (EF <35%)	−2.40	−10.20 to 5.40	0.545
Left atrial dilatation > 45 mm	0.10	−1.76 to 1.96	0.914
ACEI/ARB/AT2 antagonist	−0.44	−1.61 to 0.73	0.458
Statin	−0.65	−2.36 to 1.06	0.452
Digoxin	2.03	−3.47 to 7.52	0.468
Class I	–	–	–
Class II	−0.30	−1.68 to 1.07	0.665
Class III	−0.15	−4.66 to 4.35	0.946
Class IV	0.24	−2.27 to 2.75	0.849

β: regression coefficient; Ref: reference; BB, Bachmann’s bundle; U-AEGMs, unipolar atrial electrograms; SPs, single potentials; SDPs, short double potentials; LDPs, long double potentials; FPs, fractionated potentials; OR, odds ratio; CI, confident interval; BMI, body mass index; ACEI, angiotensin-converting enzyme inhibitors; ARB, angiotensin receptor blockers; AT2, angiotensin type 2 receptor.

**Figure 3.**
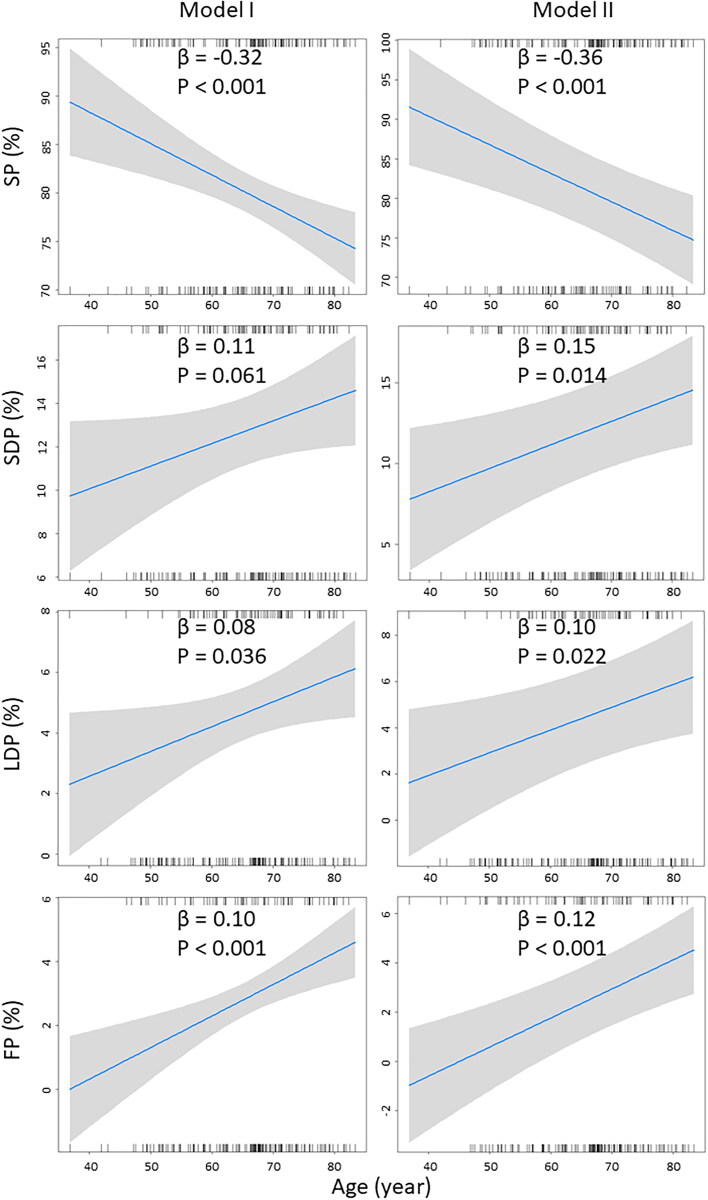
Relationship between age and BB U-AEGMs. Age is negatively correlated with SPs, and positively correlated with LDPs and FPs in model I. After adjusting for all confounding factors in model II, age is negatively correlated with SPs and positively correlated with SDPs, LDPs and FPs. Variables related to U-AEGM morphology in univariable linear regression analysis were adjusted in model I; body mass index, gender, hypertension, dyslipidemia, diabetes mellitus, myocardial infarction, left ventricular function, left atrial dilatation, and medications were adjusted in model II. U-AEGM: unipolar atrial electrogram; SP: single potential; SDP: short double potential; LDP: long double potential; FP: fractionated potential; BB: Bachmann’s bundle.

Variables introduced in the multivariable linear regression model of age-related changes U-AEGM morphology at BB are shown in Table 4. In model I, variables associated with different U-AEGMs morphologies (*p* < 0.05) in univariable linear regression were adjusted. Age was associated with a reduction in the proportion of SPs (β = −5.50, *p* = 0.005), and an increase in the proportions of LDPs (β = 1.78, *p* = 0.031) and FPs (β = 1.71, *p* = 0.005). No significant correlation between age and SDPs was found in model I (*p* = 0.128). The relation between age and U-AEGM morphology at BB is illustrated in the upper panel of Figure 3. After adjusting for all potential covariates in model II, age was associated with a decrease in the proportion of SPs (β = −6.33, *p* = 0.002), an increase in proportion of SDPs (β = 2.49, *p* = 0.042), LDPs (β = 1.94, *p* = 0.028) and FPs (β = 1.90, *p* = 0.004). The independent relationship between age and BB U-AEGM morphology is shown in the lower panel of Figure 3.

**Table 4.** Independent relationship between aged (independent variable) and BB U-AEGMs morphologies (dependent variables).

Independent variable	Dependent variables	β	95% CI	*p* Value
		**Model I**		
Aged	SPs	−5.50	−9.32 to −1.68	0.005
	SDPs	1.79	−0.52 to 4.11	0.128
	LDPs	1.78	0.16 to 3.39	0.031
	FPs	1.71	0.52 to 2.89	0.005
		**Model II**		
Aged	SPs	−6.33	−10.37 to −2.30	0.002
	SDPs	2.49	0.09 to 4.89	0.042
	LDPs	1.94	0.21 to 3.68	0.028
	FPs	1.90	0.62 to 3.18	0.004

β: regression coefficient; BB: Bachmann’s bundle; U-AEGMs: unipolar atrial electrograms; SPs: single potentials; SDPs: short double potentials; LDPs: long double potentials; FPs: fractionated potentials; OR: odds ratio; CI: confident interval; BMI: body mass index; ACEI: angiotensin-converting enzyme inhibitors; ARB: angiotensin receptor blockers; AT2: angiotensin type 2 receptor.

Variable(s) associated with different U-AEGMs morphologies in univariable linear regression was (were) adjusted in model I;

BMI, gender, hypertension, dyslipidemia, diabetes mellitus, myocardial infarction, left ventricular function, left atrial dilatation, ACEI/ARB/AT2 antagonist, Statin, Digoxin, Class II, Class III and Class IV were all adjusted in model II.

## Discussion

### Key findings

This is the first study exploring the relation between age and morphology of U-AEGMs obtained from both atria including BB. For this purpose, we used a large sample of 2,007,403 U-AEGMs. Ageing resulted in a substantial alteration in U-AEGM morphology at BB, manifested by a reduction in the proportion of SPs and an increase in proportion of SDPs, LDPs and FPs. However, ageing had no significant effect on U-AEGM morphology recorded from the RA, PVA, and LA.

### Regional differences in age-related EGM morphology

Double potentials (including SDPs and LDPs) and FPs are frequently associated with areas of conduction delay and/or block [12–14] which play a fundamental role in the pathophysiology of atrial tachyarrhythmias such as AF. Significant alterations in EGM morphology with increasing age have been demonstrated in previous studies, although these only focused on the RA. Roberts-Thomson et al. [15] categorized 21 patients without a history of AF into three groups (age < 30 years [*N* = 7]; 31 < age < 59 years [*N* = 6]; age > 60 years [*N* = 8]), and compared the proportion of bipolar complex fractionated atrial EGMs (CFAE) during SR in the RA. Their results demonstrated that the proportion of CFAE in the oldest patients was significantly higher compared to the youngest patients (14.6 ± 7.7% vs 2.7 ± 2.1%, *p* = 0.001), but no difference was observed compared to the middle group (8.5 ± 3.5%, *p* = 0.14).

Unlike the study of Roberts-Thomson et al. [15], our study did not show an influence of ageing on the U-AEGM morphology at the RA. However, as the youngest group in the study of Roberts-Thomson et al. [15] was much younger than our young group, only the middle and oldest groups can be compared to our results. Then, indeed, no difference could be found in the proportion of CFAE with age, which is consistent with the results of our present study. It should still be noted that Roberts-Thomson et al. [15] used bipolar endocardial EGMs compared to our unipolar epicardial EGMs. As Van der Does et al. [16] demonstrated that there are no differences between endo- and epicardial U-AEGM morphology recorded at the RA, our findings can still be extrapolated to endocardial U-AEGMs.

In another study of 106 patients without a history of AF, Centurion et al. [17] indicated that the number of abnormal atrial EGMs (defined as fractionation duration ≥100 ms and/or ≥8 negative deflections) in the RA during SR was considerably higher in patients over 60 years compared to younger (13 to 60 years) patients (0.61 ± 1.43 vs 0.14 ± 0.44, *p* < 0.02). The age of the patients in the young group in the study of Centurion et al. [17] was also considerably younger compared to our young group (age range from 37 to 60 years). It could therefore be that the largest differences in EGM morphology at the RA can only be found at an earlier age than included in our present study. This implies that the largest part of age-related remodeling occurs at a younger age (before approximately 50 years) after which it gradually continues, which may explain why in the present study there were no significant alterations in U-AEGM morphologies at RA (and also LA and PVA).

However, we investigated for the first time BB and found that ageing was associated with considerable changes in U-AEGM morphology at this site. This observation suggests that BB is extra vulnerable for age related remodeling. As prior studies already suggested that BB plays an important role in the pathophysiology of AF [18,19], our observations may partly explain why elderly are more prone to develop atrial tachyarrhythmia such as AF.

### Age related structural remodeling

Spach et al. [20] demonstrated that extensive collagenous septa caused by ageing resulted in electrical uncoupling of the side-to-side connections at RA, which in turn promotes variability in wave propagation direction and the complexity of EGMs. As a consequence, age-related electrophysiological alterations resulted in significant reduction in conduction velocity of transverse propagation, which makes reentry more likely to occur. Xu et al. [21] investigated the influence of ageing on atrial cellular properties in an *in-vivo* rat model, and demonstrated that the ratio of interstitial fibrotic areas to atrial surface area (2.1 ± 0.6% vs 1.0 ± 0.3%, *p* < 0.05) and cellular diameter (5.3 ± 1.1 μm vs 4.1 ± 0.8 μm, *p* < 0.05) at the LA were significantly higher in middle-aged rats (9 months) compared with those measured from young rats (3 months), but not at the RA. In a canine model, Anyukhovsky et al. [22] found that dogs older than 8 years had more connective tissue (8.4 ± 1.0% vs 4.8 ± 1.1%, *p* < 0.05) in the RA compared with dogs of 1–5 years. In addition, they found that large strands of connective tissue separated muscle bundles of elderly atrial tissue into smaller components. In our study, abnormal atrial EGMs related to ageing occurred especially at BB, indicating that age-related remodeling particularly affects BB. This observation could indicate that the parallel oriented muscle bundles contained within BB are more easily disrupted by structural remodeling. However, this hypothesis still needs further verification by subsequent histopathological studies.

### Study limitations

Morphology of U-AEGMs was not repeatedly measured at different ages of each individual patient. Therefore, the influence of individual heterogeneity cannot be excluded. Due to the invasive characteristics of intraoperative mapping, it was not possible to include patients of all ages. In addition, it cannot be completely excluded that various occlusion sites affect the atria differently. However, it is expected that the influence of coronary stenosis is comparable between the young and aged group.

## Conclusions

Ageing affects particularly BB as indicated by the increased amount of abnormal U-AEGM (SDPs, LDPs and FPs) recorded from this location in the elderly. This observation further supports the concept that BB may play an important role in development of age-related arrhythmias such as AF.

## Supplementary Material

Supplemental Material

## Data Availability

The data underlying this article will be shared on reasonable request to the corresponding author.
