# Supplementary material for: Unipolar atrial electrogram morphology is affected by age: evidence from high-resolution epicardial mapping
Source: Ann Med. 2023 May 17;55(1):1431–41. doi: 10.1080/07853890.2023.2193426 (PMC10193902; doi:10.1080/07853890.2023.2193426)
Supplement: Supplemental Material [file IANN_A_2193426_SM5257.docx]

Supplemental table 1 Characteristics of U-AEGMs for the entire study population

| Location | Total | Number of SPs (%) | Number of SDPs (%) | Number of LDPs (%) | Number of FPs (%) |
| --- | --- | --- | --- | --- | --- |
| RA | 938,093 | 773,786 (82.49) | 93,179 (9.93) | 49,288 (5.25) | 21,840 (2.33) |
| BB | 227,465 | 181,428 (79.76) | 28,478 (12.52) | 11,655 (5.12) | 5,904 (2.60) |
| PV | 445,334 | 372,207 (83.58) | 53,736 (12.07) | 12,623 (2.83) | 6,768 (1.52) |
| LA | 396,511 | 324,263 (81.79) | 50,849 (12.82) | 13,561 (3.42) | 7,838 (1.97) |

U-AEGMs, unipolar, atrial electrograms; SPs, single potentials; SDPs, short double potentials; LDPs, long double potentials; FPs, fractionated potentials; RA, right atrium; BB, Bachmann’s bundle; LA, left atrium; PV(A), pulmonary vein (area).
